# A Secure Operational Model for Mobile Payments

**DOI:** 10.1155/2014/626243

**Published:** 2014-10-20

**Authors:** Tao-Ku Chang

**Affiliations:** Department of Computer Science and Information Engineering, National Dong Hwa University, Hualien 97401, Taiwan

## Abstract

Instead of paying by cash, check, or credit cards, customers can now also use their mobile devices to pay for a wide range of services and both digital and physical goods. However, customers' security concerns are a major barrier to the broad adoption and use of mobile payments. In this paper we present the design of a secure operational model for mobile payments in which access control is based on a service-oriented architecture. A customer uses his/her mobile device to get authorization from a remote server and generate a two-dimensional barcode as the payment certificate. This payment certificate has a time limit and can be used once only. The system also provides the ability to remotely lock and disable the mobile payment service.

## 1. Introduction

Mobile devices are almost ubiquitous, their computational power is rapidly increasing, and they are as connected as personal computers or laptops. Gartner estimated that during 2013 mobile phones would replace personal computers as the most common web access device [1], while Forrester predicted that in the same year 48 percent of all US mobile-phone subscribers would be using smartphones, a striking increase from just 7 percent in 2008 [2]. A growing number of customers can use their mobile phones as keys, cameras, and TVs, and their use as a payment tool would further add to this convenience. A mobile payment has been defined as “any payment where a mobile device is used in order to initiate, activate, and/or confirm this payment” [3]. Although large-scale mobile payment systems are still under development, several mobile financial and mobile commerce applications (e.g., the Starbucks app, iTunes, and Google Wallet) are helping to increase user experiences and encourage the adoption of mobile payments among customers.

Customers usually pay for their commodities with a prepaid card or credit card in supermarkets. Many prepaid cards issued by specific stores cannot be identified when they are lost, and anyone who picks up a lost prepaid card can use it without being caught. Since shop cashiers seldom check the signature, people are usually not aware if small amounts of money are withdrawn from the balance of a lost credit card if they do not check their accounts regularly. Mobile payment technology represents an alternative payment method that can provide benefits to both customers and merchants [4, 5]. Depending on the amount of the payment, electronic payments can be classified into macro- and micropayments. Macropayment schemes are used by most e-commerce websites, and they use complex encryption techniques to achieve more rigorous security requirements [6, 7]. In contrast, micropayment schemes only need low-overhead hashing functions and are suitable for specific mobile commerce applications associated with low-value and high-volume purchases [8, 9]. However, implementing secure solutions with authentication, nonrepudiation, and privacy is still a major problem. The high cost and complicated configurations also limit the development of mobile payments. All previous payment schemes provided solutions that did not consider the factors of time and location. This situation motivated us to design an operational model that is secure, inexpensive, and convenient.

The remainder of this paper is organized as follows: Section 2 presents the secure operational model for mobile payments, Section 3 presents our implementation of the model and experience results, Section 4 gives an overview of related work and technologies, and Section 5 draws conclusions about the work described in the paper.

## 2. The Proposed Secure Payment Model

As an added security measure, a customer can request alerts for various types of account activities, such as text message for each transaction and transactions exceeding preset limits. We propose an operational model for ensuring the security of payments made with mobile devices. A fine-grained access authorization control is added to the proposed system. The business scenario and operating procedure are described in Sections 2.1 and 2.2, respectively.

### 2.1. The Business Scenario in Mobile Payments

**Figure 1:**
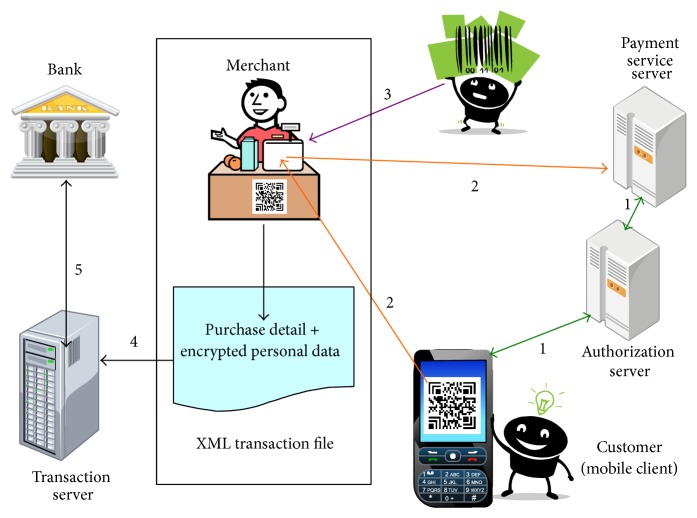
The business scenario of the payment mechanism.

Figure 1 shows the scenario for the proposed payment model. The following steps are involved when a customer wants to make a payment.

1. The customer executes the authorization application provided by a bank, which allows him or her to input the account username and password. The application then connects to the authorization server of the bank to check whether this customer is authorized to use this service. If access permission is granted, the authorization server executes a two-dimensional (2D) barcode [10] certificate service and responds with a small amount of essential data such as an authorization number and amount limit that the application uses to generate a 2D barcode.
2. The customer shows the 2D barcode on the mobile device to the cashier so that it can be scanned. The 2D barcode is decoded by the checkout system to retrieve essential data and verify the signature to confirm that the data are valid.
3. The cashier uses a scanner to scan the barcodes on goods bought by the customer. The total price and transaction data are stored in an XML data format.
4. Personal data and purchase details are stored into an XML transaction file and then transferred to the server. The personal data will be encrypted to protect it from disclosure.
5. The transaction data in the transaction server will be sent to the bank to allow a settlement process to be performed at a certain time.

A customer is involved in steps 1–3 of this payment mechanism, while steps 4 and 5 involve communication between the stores and banks. All of the components of this architecture are described in Sections 2.2–2.5.

### 2.2. Operating Procedure of the Proposed Model

**Figure 2:**
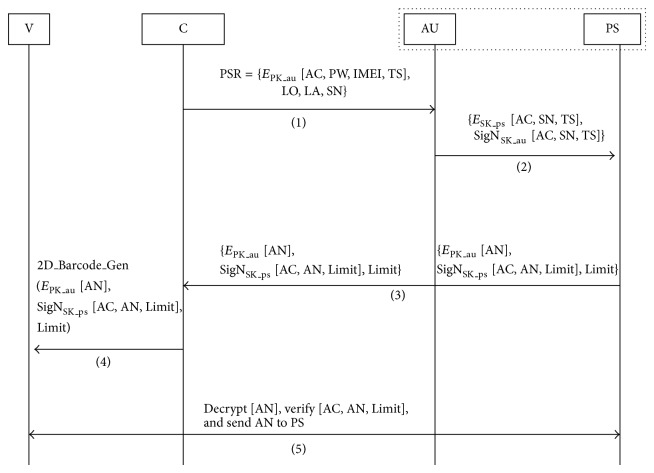
The message exchanges of the operating procedure.

The proposed model comprises the following entities: customer, vendor, authorization server, and payment-service server. The authorization server, payment-service server, and vendor have the following public and private key pairs: (PK_au, SK_au), (PK_ps, SK_ps), and (PK_v, SK_v), respectively. The details of the message exchanges of the operating procedure illustrated in Figure 2 are as follows.

1. The customer sends {E_PK_au_ [AC, PW, IMEI, TS], LO, LA, SN} to the authorization server: the authorization application provides an interface that allows customers to input the account username and password and then sends a request containing this information to the authorization server.
2. The authorization server sends {E_PK_ps_  [AC, SN, TS], SigN_SK_au_  [AC, SN, TS]} to the payment-service server: the server provides the service of verifying if users have authorization to use the 2D barcode certificate service.
3. The payment-service server sends {E_PK_au_  [AN], SigN_SK_ps_  [AC, AN, Limit], Limit} to the authorization server and the customer: after access permission is granted, the server executes the service of generating a payment certificate and responds by providing the customer's identification data to the customer.
4. The customer sends {2D_Barcode_Gen (E_PK_au_  [AN], SigN_SK_ps_  [AC, AN, Limit], Limit)} to the vendor: the authorization application calls a 2D barcode encoder to generate a 2D barcode that acts as a payment certificate.
5. The vendor sends {Sig_SK_v_  [AN]} to the payment server: the vender decrypts and verifies the data stored in the 2D barcode, and then the checkout system sends this authorization number to the payment-service server to indicate that the authorization number was used,

where

 AC: customer's account username,
 PW: customer's account password,
 IMEI: International Mobile Equipment Identity,
 TS: time stamp,
 LO: longitude of the position of the mobile device,
 LA: latitude of the position of the mobile device,
 SN: service name,
 AN: authorization number,
 Limit: amount limit of a single transaction,
 E_PK_au_: encrypt using the public key of the authorization server,
 E_PK_ps_: encrypt using the public key of the payment server,
 SigN_SK_au_: sign using the private key of the authorization server,
 SigN_SK_ps_: sign using the private key of the payment server,
 SigN_SK_v_: sign using the private key of the vendor.

This payment certificate has a time limit: if the customer does not use it within this time limit, the 2D barcode will lose its validity. Moreover, the code can be used once only. To ensure that security requirements are satisfied, these data could be encrypted and signed before being encoded. A secure communication protocol such as the HTTPS (Hypertext Transfer Protocol Secure) could be used for transferring data from the server to the mobile devices.

### 2.3. Access-Control Model of the Authorization

**Figure 3:**
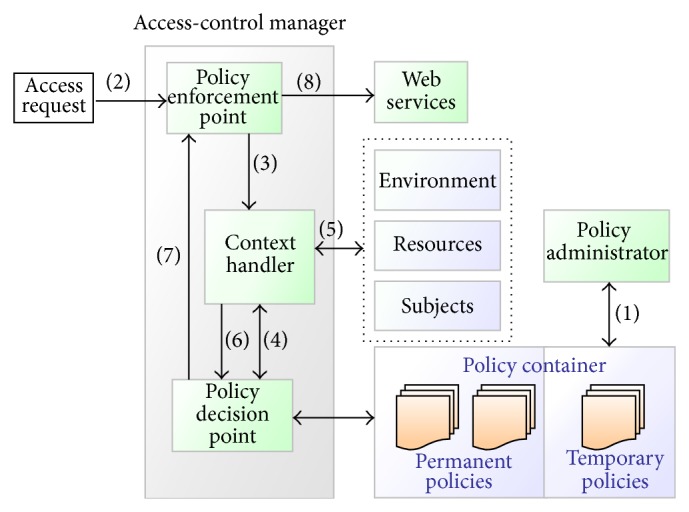
Access-control model.

The access control is handled by the access-control manager service, and the security policy is defined and stored in the policy container. We consider that the authentication policy must be decided according to the running state. A user can also grant authority to another person to build a temporary policy, such as an additional credit card. The access-control manager refers to the temporary and permanent security policies to decide if a particular request is accepted or denied. This access-control model is also appropriate for cloud-based computing. According to the data-flow model of XACML [11], we designed the access-control model depicted in Figure 3. The model operates by the following steps.

1. All of the policies stored in the policy container represent the complete policy for a specified target. The policy administrator writes these policies and makes them available when evaluating whether or not access should be allowed.
2. The customer sends an access request to the policy enforcement point (PEP) for a web service.
3. The PEP sends the request for access to the context handler in its native request format.
4. The context handler constructs an XACML request context and sends it to the policy decision point (PDP). The PDP requests any additional subject, resource, environment, and other attributes from the context handler.
5. The context handler requests the attributes from subjects, resources, and the environment.
6. The context handler sends the requested attributes and the resources to the PDP.
7. The PDP evaluates the policy and returns the response to the PEP.
8. If access is permitted, the PEP permits access to the resource and sends the client's request to the web service (i.e., payment service).

In this security mechanism, a customer can preset the effective duration scope of using the service or the location of a supermarket where he or she normally uses the service. These settings are written in customer's XACML policy.

### 2.4. 2D Barcode Encoder/Decoder

**Figure 4:**
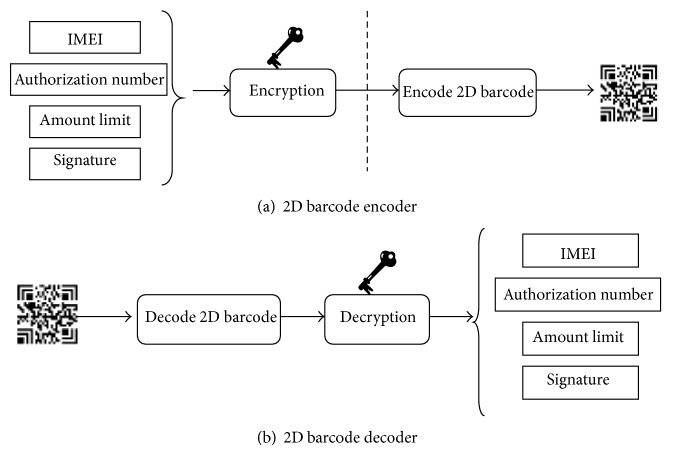
2D barcode encoder/decoder.

A 2D barcode encoder/decoder has been implemented for mobile devices and checkout systems. The encoder is called by the authorization app to generate a 2D barcode, while the decoder is invoked when a 2D barcode is read from a mobile device by a barcode scanner of the checkout system.  Figure 4 shows the processing model of the 2D barcode encoder/decoder. The data from the payment server that are embedded in the 2D barcode comprise essential information, such as the IMEI (International Mobile Equipment Identity) number of the device, an authorization number, amount limit, and signature, and could be encrypted before being encoded. Furthermore, the 2D barcode generated on the server side or the client site is also an issue. For performance considerations, we chose to generate the 2D barcode on the client device.

### 2.5. XML Securing Tool and Document Security Language

Sometimes purchase details are sensitive and important, such as a personal ID or account number, and they must be protected from even the database administrator. An XML securing tool is therefore needed to secure data because the transaction and personal information are formatted in XML. We applied XML encryption and signature technology to achieve the security requirements and defined a document security language (DSL) that describes how to encrypt and sign an XML document [12]. The structure of a DSL document can be divided into five sections: key definition, algorithm definition, security pattern, signature definition, and transformation description section. A DSL document defines the transformation description for encrypting and decrypting and embedding and verifying signatures. It offers a security mechanism that integrates element-wise encryption and temporal-based element-wise digital signatures. The temporal-based element-wise digital signature model provides a flexible way to construct and embed digital signatures in the secured document. The model is element-wise because the signed data are the collection of elements or content of elements from the source XML document, and it is temporal-based because the signature can be constructed and embedded either before or after encrypting the XML document.

**Figure 5:**
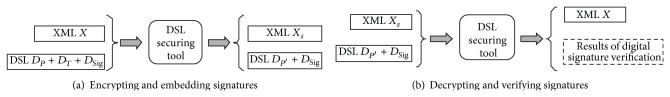
The process of securing an XML document.

Figure 5 illustrates the relationship between XML, DSL, and the* DSL securing tool*.  Figure 5(a) shows the process of encrypting and embedding digital signatures. The encryption and digital signature details are stored in a DSL document in *D*_*P*_, *D*_*T*_, and *D*_Sig_: *D*_*P*_ is the security pattern definition that specifies the combination of security algorithms and encryption and decryption keys, *D*_*T*_ is the transformation description definition that specifies the actual data transformation of element-wise encryption, and *D*_Sig_ specifies how to embed digital signatures in the resulting XML document. The target XML document that is ready to be encrypted and signed is *X*. The DSL securing tool reads, parses, analyzes *D*_*P*_, *D*_*T*_, *D*_Sig_, and *X*, and then generates *X*_*s*_ and *D*_*P*′_. *X*_*s*_ is still an XML document, but some of its elements contain ciphertexts that are translated by the DSL securing tool according to the encryption details recorded in *D*_*P*_ and *D*_*T*_. In addition to the encrypted elements, *X*_*s*_ also contains signatures that are embedded by the DSL securing tool. Each signature signs a portion of the data in *X*. It should be noted that *D*_*P*_ and *D*_*P*′_ may actually contain different information: *D*_*P*_ holds information describing how to encrypt *X*, whereas *D*_*P*′_ should include details of how to decrypt *X*_*s*_.  Algorithm 1 is an example of a DSL document whose details are shown in [13].

## 3. Implementation and Experimental Results

**Figure 6:**
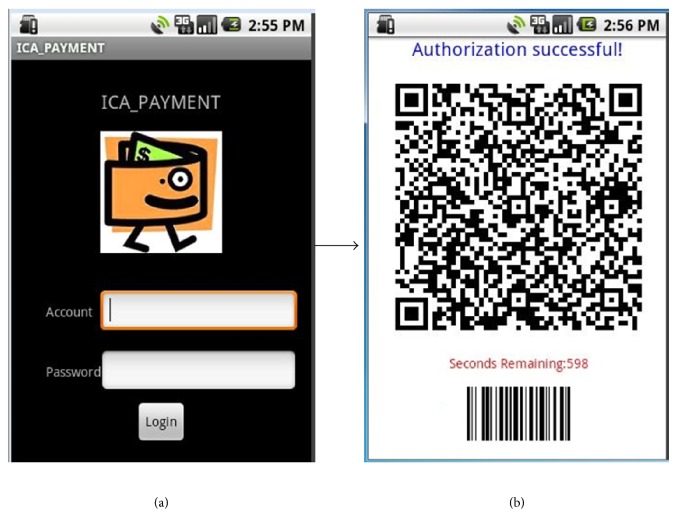
The authorization app (a) and certificate (b).

In this study we implemented each of the components described in Section 2. The authorization and the payment services were developed on the Java platform and Apache Axis2 [14]. SOAP (Simple Object Access Protocol) and Quick Response Codes (QR Codes) [15] were used as the communication protocol and the payment certificate, respectively. Customers must register before using the system, and they can temporarily and remotely stop their access right to generate a QR-Code certificate when they want to stop using this service. The authorization application, which was designed based on the Android platform, allows customers to input their account username and password to obtain the authorization for generating a QR Code as the payment certificate (see Figure 6(a)). In this implementation, the duration of the QR Codes can be set to a minute countdown by the customer (see Figure 6(b)). The barcode on the bottom of Figure 6(b) is the password that decrypts the data from the authorization server.  Algorithm 2 shows the request and response SOAP messages. Essential data are encrypted and signed to ensure the security.

**Figure 7:**
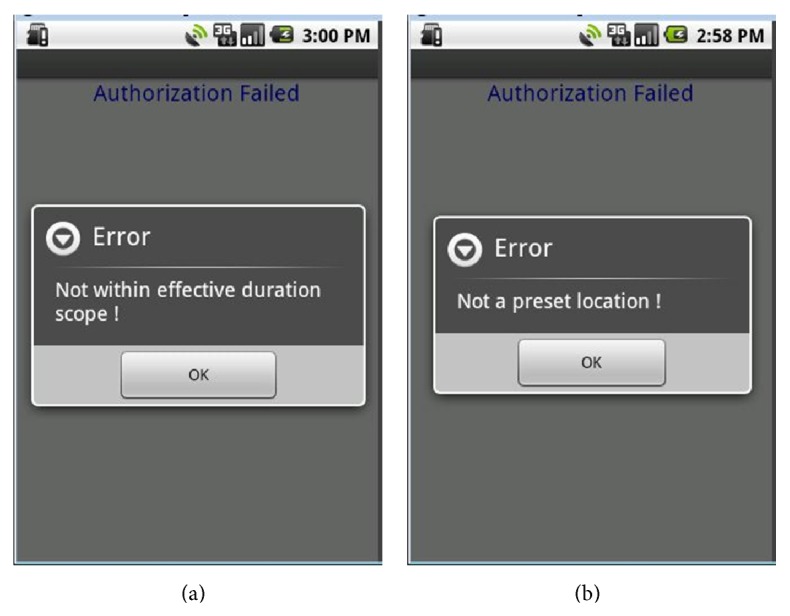
Examples of authorization failure.

**Figure 8:**
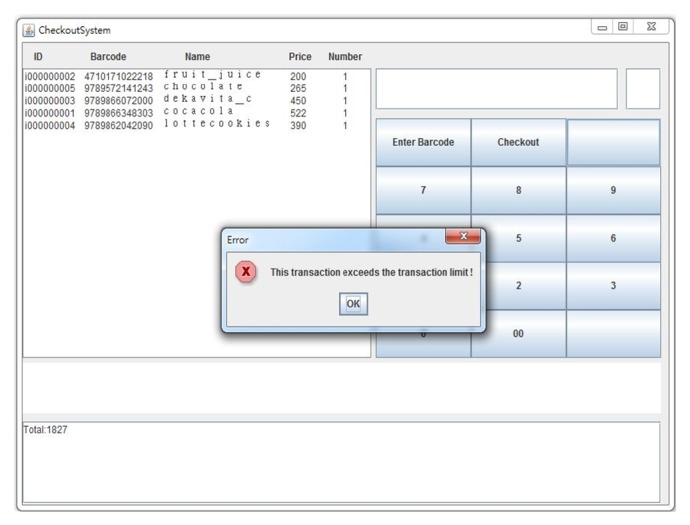
An example of a transaction exceeding the preset limit.

In this security mechanism, a customer can preset the effective duration scope of using the service or the location of a supermarket where he or she normally uses the service. These settings are written in customer's XACML policy (see Algorithm 3). The QR-Code certificate service is disabled when its attempted use isnot in a valid duration scope or a designated location. For example, the authorization fails on April 25, 2014, when the effective duration scope is from January 1 to March 31, 2014 (see Figure 7(a)), and when attempting to use the service in Hualien City when the designated location is set to be in Taipei City (see Figure 7(b)). A customer can also preset an amount limit for a single transaction, in which case the monitor of the checkout system sends out an error message when this transaction exceeds the amount (see Figure 8).

After a customer has finished a transaction, the details of the transaction are formatted in XML and some important data are encrypted.  Algorithm 4 shows an example of the encrypted data of a purchase. In this case only the authorization number is encrypted, and the encryption key is encrypted by the public key of the bank.

We conducted experiments with JMeter to evaluate the performance of the proposed model [16]. All the experiments were performed on a PC with a 2.8 GHz Intel-i7 quad-core processor, 2 GB of RAM, the MS Windows 7 operating system, and Java Development Kit 7 update 45.

We compared the times required to secure the message between the client and the server using the SSL protocol (Tables 1 and 2) and the RSA cipher (Tables 3 and 4). This comparison was based on the times required to submit different numbers of requests at the same time within 1 second and 10 seconds. The experimental results indicate that the performance was better when using the SSL protocol than when using an RSA cipher. It was also clear that the performance was improved dramatically when using two Tomcat servers.

## 4. Security Analysis

In this paper we assume that the authorization server and the payment server are honest and are trusted by both the customer and vendor. However, the customer and vendor may or may not be honest. The main goal of the proposed model is to determine the authenticity of the payment certificate and to prevent the problems of counterfeiting and reusing the certificate. These features were implemented as follows.

1. Confidentiality and authentication: the proposed model employs the SSL protocol to authenticate the server and to cryptographically protect the communication channel between the client and the server. The client must provide the customer's account username and password, as well as the IMEI number and the longitude and latitude of the position of the mobile device, to the authorization server. An attacker can guess the account and password. However, he cannot get the mobile device to obtain an IMEI number.
2. Attacker-counterfeit and customer-counterfeit attacks: the information in the payment certificate, {E_PK_au_  [AN], SigN_SK_ps_  [AC, AN, Limit], Limit}, is generated by the payment server, signed, and then encrypted by the payment server's key. This means that an attacker cannot counterfeit any certificate for payment.
3. Vendor-counterfeit attack: the payment certificates, including AC, AN, and Limit, are recorded in the payment server and vendor, and so the vendor cannot counterfeit the transactions since the auditing scheme will detect inconsistency in the data between the vendor and the payment server.
4. Reuse attack: when the authentication is passed, the payment-service server sends {E_PK_au_  [AN], SigN_SK_ps_  [AC, AN, Limit], Limit} to the authorization server and the customer. Once the certificate is used, the checkout system sends the authorization number to the payment-service server to indicate that the certificate has been used. This protocol prevents a reuse attack in our proposed scheme.

## 5. Related Work and Technologies

Customers now carry mobile phones more often than a wallet or purse. The average amount of time it takes someone to realize he or she has lost a wallet is typically 5-6 hours; in contrast, it typically takes someone about 15 minutes to realize a mobile phone is missing [17]. The mobile medium is an easy environment via which promotions and market incentive services can be issued to customers. Industry analysts and service providers have identified several important drivers for the adoption of mobile payments by customers, such as familiarity and comfort with using mobile technology, strong security, and greater convenience. Security and privacy risks are major barriers to adoption, with customers worrying about their personal data being hacked or intercepted. They consider mobile transactions to be less secure than credit- and debit-card transactions, whereas mobile payments can be just as secure as or even more secure than traditional payment methods. When customers are offered a secure online payment environment, which works via advanced mobile web systems, customers do not need to provide physical currency each time they want to make a mobile purchase or pay a bill online.

Experts from various sectors contend that mobile payments will soon become very popular. Recent studies indicate that customer awareness and interest in mobile payments have been increasing. A survey performed by the Consumer Research Section of the Board of Governors of the Federal Reserve System found that more than half of customers believed that mobile contactless payments would become a major form of payment within the next 5 years, and more than one-third of the survey subjects indicated that they would use this method of payment if it were made available to them [18]. Mobile payments overall are expected to move toward the mainstream, with their value reaching $90 billion by 2017 in the US, according to a Forrester report [19].

Mobile payments can be differentiated based on various characteristics, including the technology used and the transaction size, location, and funding mechanism [20]. The technology can be categorized as either one of two types: proximity or remote. Proximity payments generally refer to contactless payments employing near-field communication (NFC) [21–23], while remote payments are made via a mobile web browser or a smartphone application, in which the mobile phone is used as a device to authenticate personal information that is stored remotely. It uses services such as SMS (short message service) to initiate or authorize payment. The funding mechanism for payments made in a mobile payment system has previously been differentiated into a bank account, credit card, and telecommunication company billing or into an account, real time, prepaid, postpaid, smart card, credit card, mobile POS, mobile wallets, and P2P payments [3, 20, 24, 25].

A survey conducted by members of the Smart Card Alliance Contactless Payment Council considered four potential mobile payments business scenarios [26, 27].

1. Operator-centric model: the mobile operator acts independently to deploy mobile payment applications to NFC-enabled mobile devices.
2. Bank-centric model: a bank deploys mobile payment applications or devices to customers and ensures merchants have the required point-of-sale acceptance capability.
3. Peer-to-peer model: an independent peer-to-peer service provider provides secure mobile payments between customers or between customers and merchants.
4. Collaboration model: this model involves collaboration among banks, mobile operators, and a trusted third party.

Each of the models described above offers one or more scenarios for implementation. The security and speed are the main concerns of stakeholders.

Privacy and security are becoming more important to customers, given the rise of mobile payments and commerce, and continue to be a major obstacle to widespread adoption. The specific security issues identified have varied between surveys. Some of the customer reservations stem from fear of payment account information being intercepted, the threat of unauthorized parties accessing personally identifiable information, and the receipt of unsolicited promotional material [18, 28]. According to a research report from Synergistics, more than half of mobile-phone owners surveyed indicated identity theft as the top concern related to making mobile payments [29]. More than half of the customers surveyed in a first data mobile payments study believed that making a payment via mobile phone was less secure than making a payment in person or with a credit or debit card [30]. Regardless of the specific reasons for these security concerns and their validity, security issues must be addressed to achieve mass adoption of mobile payments.

Many applications on smartphones are developed for use on a web-services architecture [31]. Web-service applications run over the open, untrustworthy, and unreliable Internet, which means that web-services providers must consider security issues including confidentiality, authentication, and authorization. There are numerous standards for solving these problems, such as XML encryption [32], XML signature [33], Security Assertion Markup Language [34], extensible access-control markup language (XACML) [11], and XML Key Management Specification [35]. Some attacks about XML encryption and signature have been reported [36]. However, in our proposed model they are used to guarantee that the messages are secured and not modified in transit. It does not affect the procedure of the authentication.

Some of the main technologies used in mobile commerce are NFC, mobile wallets, and QR Codes. NFC refers to a set of short-range wireless technologies typically involving connections operating over distances up to 10 centimeters. A mobile wallet is a software application loaded on a mobile phone that enables the storage of multiple payment credentials and the secure access to value-added services in order to initiate mobile payments. A QR Code is a kind of 2D symbology developed by Denso Wave (a division of Denso Corporation at the time) and released in 1994. It contains information in two dimensions, which allows it to hold considerably more information than a barcode. These technologies are changing the way customers pay for goods and services and mean that they can now use their smartphones rather than their credit cards to make payments. Many customers are now using their mobile devices to both make shop comparisons when inside stores and also actually shop for items. Many merchants and hypermarkets provide mobile applications or QR Codes for customers to extend their markets to mobile shopping. There are now many situations in which a mobile device can be used as a payment tool, with QR Codes acting as a bridge between product information and customers. Some examples of these are as follows.

1. Starbucks card mobile is a three-part system that includes 2D barcodes, scanners, and mobile-phone applications for iPhone, BlackBerry, and HTC. This system allows Starbucks customers to pay with their phones at roughly 9,000 locations in the US. It has also helped the coffee company become the only large-scale provider of mobile payments [37].
2. Amazon provides an augmented-reality app called Flow [38] that lets customers discover information about products by scanning their QR Codes. Flow enables Amazon customers to identify tens of millions of products, including books, DVDs, and packaged household items such as boxes of cereal and boxes of tissues. The app can also quickly scan and dial phone numbers or launch websites.
3. Google launched its Google Wallet app in 2011 [39]. Customers can use any VISA, MasterCard, American Express, or Discover credit card in conjunction with the app to pay in store by simply tapping a smartphone against any contactless point-of-sale terminal. Payment information is transmitted via NFC, and shortly thereafter a transaction record with the merchant name and dollar amount appears on the phone. Google Wallet is claimed to be both safe and secure. The app has its own PIN, and it can be remotely disabled if the phone is lost.
4. EasyCard is Taiwan's most popular contactless payment service that is widely used by people using the subway, shopping, or dining. EasyCard features a graphical user interface that users allow to easily perform several functions such as balance checking, micropayment transactions, and e-wallet top-up via their Android smartphones [40].
5. 7-NET also provides QR-Code-based shopping in Taiwan. Customers can use smartphones to scan QR Codes in product advertisements, then connect to the 7-NET website to check out and take the products, and pay for them at a nearby 7-Eleven store. This represents the first example of mobile commerce being used in the convenience-store industry in Taiwan.

All previous payment schemes discussed in this paper provided solutions that did not consider the factors of time and location. In our security mechanism, a customer can preset the effective duration scope of using the service or the location of a supermarket where he or she normally uses the service. Furthermore, the payment certificate is generated and is different for each transaction and does not include sensitive data such as credit-card number.

## 6. Conclusion

Mobile payments can be made more secure than traditional payment methods. The mobile device must be set up correctly with risk-mitigation tools having the ability to remotely wipe, delete, lock, and disable a lost or stolen mobile phone, with antivirus and malware software, and the customer must use the mobile payments capabilities correctly. Furthermore, consumers must understand that they also have responsibilities to protect their payment account credentials and mobile devices. Customers also need to be educated about what not to do, such as downloading untested, questionable, or uncertified applications or sharing their mobile phones. Moreover, many or even most mobile phones will have keyboard loggers installed, in the form of predictive keyboards, many of which can communicate with remote servers due to the permissions granted to these apps when they are installed on their phones. Even if these are “certified” apps, they represent a fundamental problem with the security of mobile phones. Customers must be aware of this kind of risks. This paper has presented a secure operational model for mobile payments, in which a customer uses the mobile device to obtain authorization and generate QR Codes as the payment certificate and has multiple layers of security to lock both the phone and access to the secure mobile payment. The proposed model includes not only authentication, confidentiality, and integrity, but also nonrepudiation and privacy. The encryption and decryption processes are not performed on mobile devices, which eliminate the problem of some mobile devices being unable to support powerful cryptographic algorithms. The proposed platform based on a web-services architecture is extensible, inexpensive, and easy to configure.

## Figures and Tables

**Algorithm 1:**
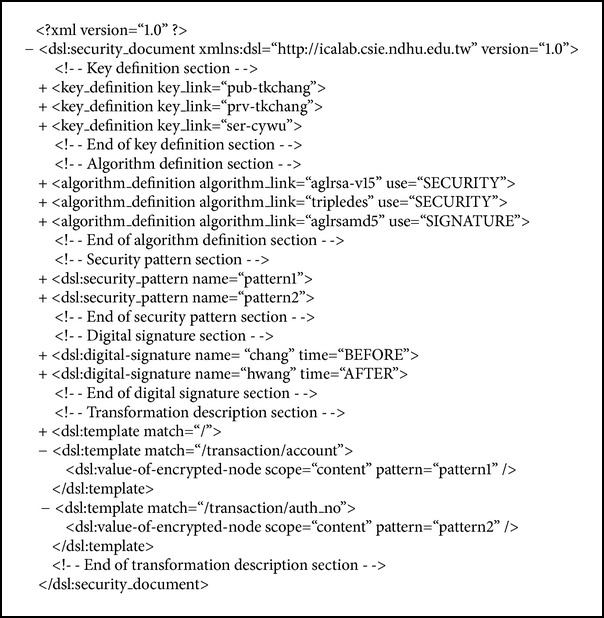
An example of a document security language.

**Algorithm 2:**
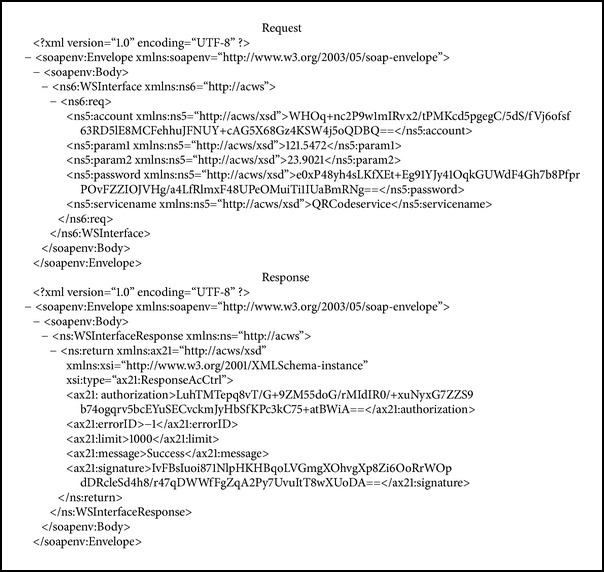
The request and response SOAP messages.

**Algorithm 3:**
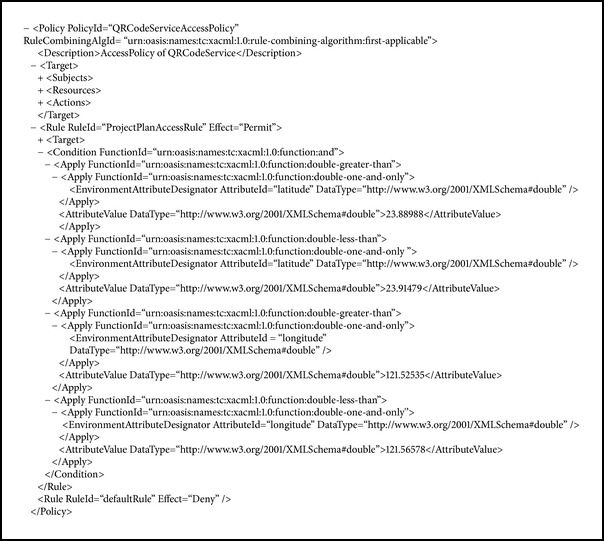
An example of location policy.

**Algorithm 4:**
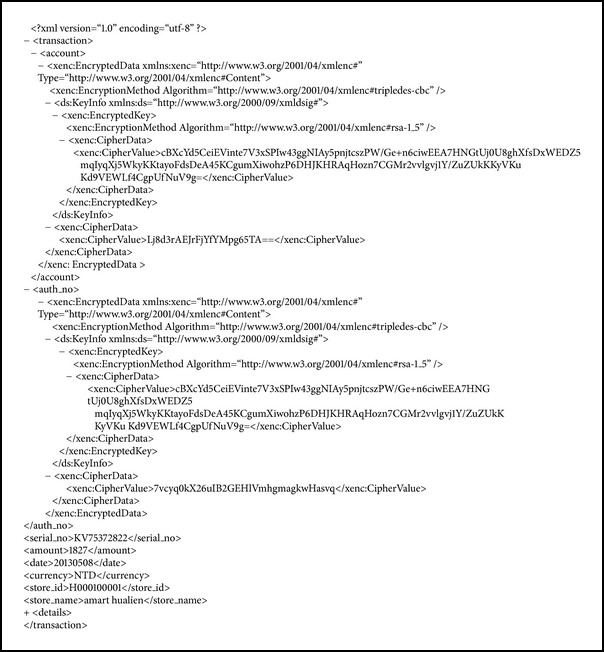
An example of encrypted data in a purchase.

**Table 1:** Times required to submit different numbers of requests within 1 second when using the SSL protocol.

Number of requests	One Tomcat server		Two Tomcat servers	
	Mean time (seconds)	Total time (seconds)	Mean time (seconds)	Total time (seconds)
1	1.703	1.703	1.943	1.943
10	1.500	15	1.100	11
20	1.500	30	1.100	22
30	1.533	46	1.033	31
40	1.525	61	1.025	41
50	1.520	76	1.040	52
60	1.383	83	1.150	69
70	1.657	116	1.043	73
80	1.475	118	1.038	83
90	1.511	136	1.167	105
100	1.520	152	1.070	107

**Table 2:** Times required to submit different numbers of requests within 10 seconds when using the SSL protocol.

Number of requests	One Tomcat server		Two Tomcat servers	
	Mean time (seconds)	Total time (seconds)	Mean time (seconds)	Total time (seconds)
1	1.661	1.661	1.994	1.994
10	1.300	13	1.200	12
20	1.500	30	1.100	22
30	1.400	42	1.133	34
40	1.400	56	1.250	50
50	1.380	69	1.140	57
60	1.367	82	1.150	69
70	1.371	96	1.157	81
80	1.400	112	1.275	102
90	1.489	134	1.267	114
100	1.600	160	1.170	117

**Table 3:** Times required to submit different numbers of requests within 1 second when using an RSA cipher.

Number of requests	One Tomcat server		Two Tomcat servers	
	Mean time (seconds)	Total time (seconds)	Mean time (seconds)	Total time (seconds)
1	3.163	3.163	3.598	3.598
10	3.000	30	2.000	20
20	3.000	60	2.000	40
30	2.967	89	2.000	60
40	2.950	118	2.025	81
50	2.980	149	2.060	103
60	2.967	178	2.017	121
70	2.986	209	2.014	141
80	3.338	267	2.238	179
90	3.178	286	2.000	180
100	3.030	303	2.060	206

**Table 4:** Times required to submit different numbers of requests within 10 seconds when using an RSA cipher.

Number of requests	One Tomcat server		Two Tomcat servers	
	Mean time (seconds)	Total time (seconds)	Mean time (seconds)	Total time (seconds)
1	3.168	3.168	3.557	3.557
10	2.900	29	2.000	20
20	3.000	60	2.000	40
30	2.933	88	2.233	67
40	2.975	119	2.225	89
50	3.000	150	1.980	99
60	2.983	179	2.017	121
70	3.029	212	1.857	130
80	3.050	244	2.288	183
90	3.000	270	2.078	187
100	3.070	307	2.010	201
